# Interaction Between Halotolerant Phosphate-Solubilizing Bacteria (*Providencia rettgeri* Strain TPM23) and Rock Phosphate Improves Soil Biochemical Properties and Peanut Growth in Saline Soil

**DOI:** 10.3389/fmicb.2021.777351

**Published:** 2021-12-16

**Authors:** Huanhuan Jiang, Sainan Li, Tong Wang, Xiaoyuan Chi, Peishi Qi, Gang Chen

**Affiliations:** ^1^College of Life Sciences, Zhaoqing University, Zhaoqing, China; ^2^Shandong Peanut Research Institute, Qingdao, China; ^3^State Key Laboratory of Urban Water Resource and Environment, School of Environment, Harbin Institute of Technology, Harbin, China

**Keywords:** saline soil, rhizosphere, phosphate-solubilizing bacteria, rock phosphate, peanut growth, nutrient uptake, soil properties, bacterial community

## Abstract

Soil salinity has adverse effects on soil microbial activity and nutrient cycles and therefore limits crop growth and yield. Amendments with halotolerant phosphate-solubilizing bacteria (PSB) and rock phosphate (RP) may improve properties of saline soil. In this study, we investigated the effects of RP either alone or in combination with PSB (*Providencia rettgeri* strain TPM23) on peanut growth and soil quality in a saline soil. With the combined application of RP and PSB, plant length and biomass (roots and shoots) and uptake of phosphorus (P), nitrogen (N), and potassium (K) increased significantly. Soil Na^+^ and Cl^–^ contents decreased in the PR alone or PR combined with PSB treatment groups. There were strongly synergistic effects of RP and PSB on soil quality, including a decrease in pH. The soil available N, P, and K contents were significantly affected by the PSB treatments. In addition, the alkaline phosphomonoesterases, urease, and dehydrogenase activities increased significantly compared with the untreated group; highest alkaline phosphomonoesterases activity was observed in the RP and PSB treatment groups. The composition of rhizosphere soil bacterial communities was determined using 454-pyrosequencing of the 16S rRNA gene. In the PR alone or PR combined with PSB treatment groups, the structure of the soil bacterial community improved with increasing richness and diversity. With PSB inoculation, the relative abundance of *Acidobacteria*, *Chloroflexi*, and *Planctomycetes* increased. The three phyla were also positively correlated with soil available N and root dry weight. These results suggested microbiological mechanisms by which the combined use of RP and PSB improved saline soil and promoted plant growth. Overall, the study indicates the combined use of RP and PSB can be an economical and sustainable strategy to increase plant growth in P-deficient and salt-affected soils.

## Introduction

Saline soil is a serious global environmental problem that negatively affects agricultural production because of unfavorable soil physicochemical properties and nutrient deficiencies (Shrivastava and Kumar, 2015). Accumulation of excessive sodium ions can adversely affect soil nutrient cycles (Li et al., 2016), enzyme activity (Patel and Saraf, 2013), and microbial community diversity (Bharti et al., 2016). Phosphorus (P) is one of the most important macronutrients required for plant growth. However, most P in soil is in inorganic and organic forms with low solubility that are not available for plant uptake (Adnan et al., 2017). The low availability of P in saline soil is due to fixation when P is bound to calcium (Ca), aluminum, and iron (Tchakounté et al., 2020). Salt stress can also reduce plant uptake efficiency of P, which leads to losses in crop yields (Radhakrishnan et al., 2015). To overcome P deficiency, P fertilizers can provide adequate P to increase plant productivity, although excessive application can cause eutrophication and is not economical (Park et al., 2010). Therefore, in saline soils, management must be improved to increase the availability of P and also minimize its adverse effects. In sustainable agriculture, application of biofertilizers is one potential strategy to minimize the environmental problems associated with chemical fertilizers (Chang and Yang, 2009). The use of microbial inoculation combined with rock phosphate (RP) is recognized as a suitable strategy to improve saline soil properties and plant growth (Singh and Reddy, 2011); Phosphate-solubilizing bacteria (PSB) are an integral component of the soil P cycle and play a key role in dissolving different pools of soil P to increase P availability to plants. Many PSB can significantly increase plant growth in saline soils by releasing soluble P (Ramadoss et al., 2013; Wan et al., 2020). The most effective microbes at solubilizing P include some common genera of bacteria and fungi, such as *Pseudomonas*, *Bacillus*, *Micrococcus*, *Aspergillus*, *Penicillium*, and *Enterobacter* (Ghosh et al., 2016; Ludueña et al., 2018). Moreover, halotolerant PSB can facilitate plant adaptation and tolerance to salinity stress and subsequently improve plant growth (Vassilev et al., 2006). The main mechanism underlying inorganic phosphate mineralization is the production of low-molecular-weight organic acids. These acids acidify phosphate conjugate bases, thereby increasing their solubility as a result of the reduction of soil pH, while also chelating metals (Wei et al., 2018). In our previous work, a halotolerant PSB isolated from saline soil increased the availability of nutrients to plants, and in particular soil available P. The PSB used in that study, a strain of Providencia rettgeri, may thus have potential use as a biofertilizer to sustain the growth of peanut in salt-affected soil (Jiang et al., 2019). However, few studies have explored the potential of halotolerant PSB to dissolve multiple P sources.

Rock phosphate (RP) is a raw material used in manufacturing phosphatic fertilizers; it has been used as an alternative P source extensively due to its lower cost and as a support for sustainable agriculture (Xiao et al., 2011). However, the extremely poor solubility of RP limits its direct use as a soil amendment, especially in saline–alkaline soil. Previous studies have shown that RP can improve the physicochemical properties of saline soils and that inoculation with PSB enhances the effects (Baig et al., 2010). Adnan et al. (2020) found that compared with the application of only RP, the combination of RP and PSB increases maize P acquisition under salinity stress. Most studies now focus on using the combined application of RP and PSB to increase the growth of plants and improve soil physicochemical properties (Iqbal et al., 2019). Microbial communities are an integral part of soil ecosystems and are considered the main drivers of soil fertility and quality (van der Heijden and Wagg, 2013). However, microbial populations are often restricted by nutrient deficiencies in saline soils (García-Orenes et al., 2010). The use of PSB as bioinoculants has been shown to influence microbial communities that colonize the rhizosphere and further increase soil enzyme activity and nutrient contents (Li et al., 2014). According to Song et al. (2021), PSB enhanced the abundance of beneficial microorganisms and decreased the abundance of harmful microorganisms. PSB can secrete a growth-promoting substance or release a chemical signal to promote their own growth, thereby improving relative microbiology abundance and accelerating plant uptake of nutrients from the soil. Silva et al. (2017) reported that long-term RP fertilization also changes microbial community structure, with microorganisms related to P solubilization and acquisition increasing in abundance. Therefore, PSB can be used in the form of bioinoculants in agriculture to benefit the regulation of soil microbial communities. However, the effects of the interaction between RP and halotolerant PSB on rhizosphere microbial diversity remain poorly understood.

Peanut (*Arachis hypogaea* L.) is an economically valuable crop. However, high salt levels and P deficiency limit peanut production and ultimately lead to severe losses in yield (Sharma et al., 2016). Information on the combined application of halotolerant PSB and RP is lacking for peanuts grown in saline soils. In addition, the effects of the combined application of halotolerant PSB and RP on saline soil microbial communities have not been investigated. Our hypothesis, therefore, was that introducing PR in conjunction with PSB inoculation to saline soils would modify their properties and bacterial community. This treatment may be useful for increasing the available phosphorus content and promoting plant growth. Thus, the primary objective of this study was to evaluate the effects of the interaction between halotolerant PSB and RP on soil properties and peanut growth in a saline soil. Changes in bacterial community structure were examined to better understand the microbiological mechanisms underlying the improvements in soil properties and plant growth.

## Materials and Methods

### Bacterial Strain Culture and Seed Treatment

*Providencia rettgeri* strain (TPM23) (Accession no. KX289656.1) is a halotolerant PSB described in our previous work (Jiang et al., 2019). The tricalcium phosphate medium (TPM) [10 g glucose, 0.5 g yeast extract, 0.5 g (NH_4_)_2_SO_4_, 0.26 g KCl, 0.1 g MgSO_4_.7H_2_O, 0.2 g NaCl, 0.05 g FeSO_4_.7H_2_O, 0.2 mg MnSO_4_.H_2_O, 5 g Ca_3_(PO_4_)_2_, and 1,000 mL of distilled water] was added to flasks (200 mL). Then, 200 μL of the isolate were inoculated for 24 h at 28 ± 2°C with continuous shaking. Uninoculated medium was used as control. The bacterium suspension was adjusted to a final concentration of 10^8^ colony forming units (CFU) per mL.

The peanut cultivar Huayu33 was provided by the Shandong Peanut Research Institution (China Qingdao). Seeds were sterilized in 75% ethanol and 10% NaClO_3_ solutions for 1 and 10 min, respectively. After washing with sterilized distilled water, some seeds were cultured with the PSB isolate at 10^8^CFU/mL for 3 h. Seeds in uninoculated medium were the controls.

### Pot Experiment

Soil samples were collected from sites known to have excessive salinity in the Yellow River delta area of Dongying Prefecture, Shandong, China (lat 37.5, long 118.3). We collected 10 samples at an interval of 1 m between each sampling point. The samples were scraped off from encrusted environmental surface soils. In addition, 15-cm soil cores were collected and mixed well. After air-drying at room temperature, soils were ground to pass through a 2-mm sieve. The characterization of the experimental soil used in the current study is described in Table 1. The main compound in RP is CaSO_4_, which is acidic and contains the essential plant nutrients Ca, P, and sulfur. In addition, RP contains 15–20% P. Plastic pots (height, 25 cm; diameter, 25 cm) were filled with equal amounts (approximately 1.5 kg) of sieved soil. One group of pots contained only original soil (T0), and the other group contained original soil plus 20 g/kg RP (T1). After the soil was fully mixed, half the pots in each group were inoculated with 15 mL of PSB suspension (+PSB) at 10^8^CFU/mL, resulting in the treatments T0P and T1P. The other half in each group did not receive PSB inoculum (-PSB) but were mock-treated with 15 mL of sterilized water, resulting in the treatments T0 and T1. Five inoculated or control peanut seeds (prepared as described above) were sown in each pot (Table 2). After 7 days, the plants were thinned out to three plants per pot. The pot experiment was arranged in a two factor completely randomized design with three replicates per treatment. The experiment was conducted for 45 days in a growth chamber at 24 ± 1°C with a 16-h light and 8-h dark photoperiod (350 μmolm^–2^ s^–1^ light intensity), and a relative humidity of 50%. Samples were watered regularly with sterilized tap water twice a month.

**TABLE 1:** Soil characterization.

pH	Moisture content	TP	TN	TK	AP
	**(%)**	**(mg/kg)**	**(mg/kg)**	**(mg/kg)**	**(mg/kg)**
7.99	15.43	278.9	181.31	2300.21	9.31

*TP, total P; TN, total N; TK, total K; AP, available P.*

**TABLE 2:** Pot experiment treatment for each experimental group.

Experimental group	Plant treatment method	Soil treatment method
T0	Seeds cultured in uninoculated medium	Original soil
T0P	Seeds cultured in PSB suspension	Original soil + PSB suspension
T1	Seeds cultured in uninoculated medium	Original soil + RP
T1P	Seeds cultured in PSB suspension	Original soil + RP + PSB suspension

### Plant Growth and Yield Attributes

At maturity, peanut plants in three replicates from each treatment were harvested, and root and shoot lengths, and dry weights, as well as total branch number (TBN) and total leaf number (TLN), were determined. The roots and shoots were washed several times with sterilized distilled water. Plant material was dried at 80°C for 7 days, and dry weights were recorded. To determine total N (TN), total P (TP), and total K (TK) contents, peanut leaves were washed several times with sterilized distilled water and then oven-dried at 80°C for 48 h. Dried peanut leaves (0.1 g) were digested at 150°C for 2 h by the H_2_SO_4_-H_2_O_2_ cooking method. The content of total nitrogen was determined by the Kjeldahl method. The content of total phosphorus was determined by vanadate-molybdate-yellow colorimetry. The content of total potassium was determined by a flame photometer.

### Soil Physicochemical Properties and Enzyme Activities

After plants were harvested, soil was collected and analyzed for physicochemical properties and enzyme activities. Soil pH and electrical conductivity (EC) were determined using a pH meter (Hanna HI2221, Italy) and a conductometer (DDS-307A), respectively. Available N (AN) was extracted with 2 M KCl, and content was determined using the alkaline hydrolysis diffusion method (Mulvaney and Khan, 2001). Available P (AP) was extracted with 0.5 M NaHCO_3_, and content was determined using molybdate colorimetry (Murphy and Riley, 1962). Available K (AK) was extracted with a CH_3_COONH_4_ solution, and content was measured by a flame photometer (AA-6300C, Shimadzu Japan). The activities of alkaline phosphomonoesterases (PP), dehydrogenase (DHA), and urease (UR) were determined by using ELISA kits (Shanghai Yuanye Biotechnology Co. Ltd., Shanghai, China).

### Soil DNA Extraction and PCR Amplification

From the rhizosphere soil samples collected after peanut harvest, 2 g of soil from each replicate of the four treatments was collected. The V3–V4 region of the bacterial 16s RNA gene was amplified by PCR. After purification, PCR products were separated by electrophoresis on 2% agarose gel. Sequencing libraries were generated using a TruSeq^®^ DNA PCR-Free Sample Preparation Kit (Illumina, United States) following the manufacturer’s recommendations, and index codes were added. Library quality was assessed on a Qubit@ 2.0 Fluorometer (Thermo Scientific) and an Agilent Bioanalyzer 2100 system. The library was sequenced on an Illumina HiSeq2500 platform, and 250-bp paired-end reads were generated.

### Operational Taxonomic Unit-Based Sequence Analysis

Paired-end reads were assigned to samples based on their unique bar codes and truncated by cutting off the bar code and primer sequence. To obtain high-quality clean tags, QIIME^1^ was used to perform quality filtering on the raw tags under specific filtering conditions. The effective tags were obtained after removal of chimera sequences. Sequences with ≥97% similarity were assigned to the same operational taxonomic unit abundances (OTUs). The observed-species, Chao1, ACE, Shannon, and Simpson indices of the bacterial communities were calculated in the QIIME software. To evaluate significant differences in soil bacteria between groups with a PSB suspension (T0P, T1P) and without PSB inoculation (T0, T1), *t*-tests were used. To evaluate the possible effects of soil environmental factors on bacterial communities, Pearson’s coefficients of correlation (*r*) and significance (*P*) values were calculated between the most abundant bacterial phyla and peanut growth parameters and soil properties.

### Statistical Analyses

Two-way ANOVA (*P* < 0.05; *F*-test) and Student’s *t*-test (*P* < 0.05) were performed using SPSS v 13.0 (SPSS Inc., Chicago, IL, United States) software to determine the differences in pot experimental data among the different treatments. Differences were detected by the standard error of difference (SED).

## Results

### Peanut Growth Parameters and Macronutrient Accumulation

**FIGURE 1:**
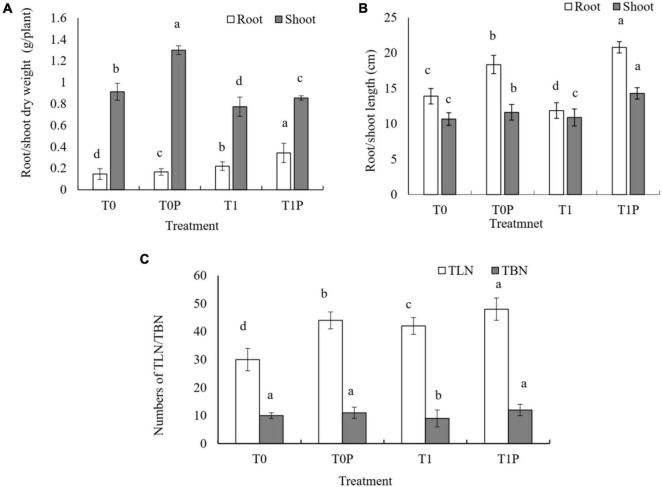
Effects of rock phosphate (RP) and phosphate-solubilizing bacteria (PSB) on peanut **(A)** root and shoot dry weight (g plant^–1^), **(B)** root and shoot length (cm), and **(C)** total leaf number (TLN) and total branch number (TBN). Treatments: T0, no additions, the control; T0P, with PSB inoculation only; T1, with RP amendment only; T1P, with RP and PSB. Values are the means of three replicates. Error bars represent ± standard deviation. Values sharing a common letter within the column are not significant at *P* < 0.05.

The effect of RP with or without PSB on peanut growth efficiency was investigated. According to the two-way ANOVA, the effects of RP, PSB, and their interaction on peanut growth measures were significant (*P* < 0.05). Peanuts inoculated with PSB had greater root and shoot lengths compared with those without PSB, with or without RP (Figure 1B). Without PSB, RP had only slight effects on peanut biomass. However, when RP was combined with PSB inoculation, shoot and root dry biomass were also significantly affected by PSB inoculation (Figure 1A). Compared with the PR only treatment, the dry weight of roots increased by 13.61 and 55.91% and the dry weight of shoots increased by 42.81 and 10.62% in T0P and T1P, respectively. The same increasing trend was observed for TLN and TBN (Figure 1C).

**FIGURE 2:**
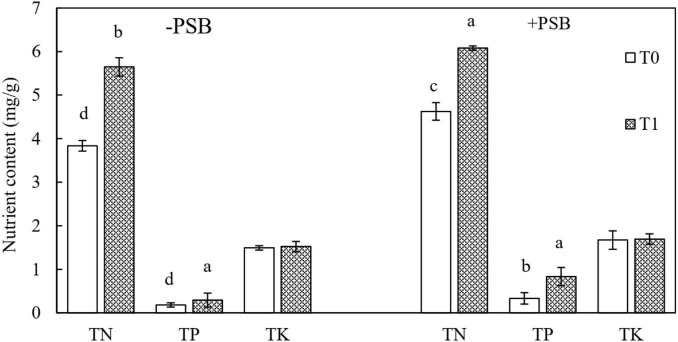
Effects of rock phosphate (RP) and phosphate-solubilizing bacteria (PSB) on total N (TN), total P (TP), and total K (TK) accumulation (mg/g) in peanut leaves. Treatments: T0, without RP; T1, with RP; −PSB, without PSB; +PSB, with PSB. Values are the means of three replicates. Error bars represent ± standard deviation. Values sharing a common letter within the column are not significant at *P* < 0.05.

Contents of TN, TP, and TK in peanut leaves are shown in Figure 2. The contents of TN and TP were different among the experimental groups with and without PSB inoculation (*P* < 0.05), but TK content was not affected (*P* > 0.05). The accumulation of nutrients was higher with PSB inoculation than without (Figure 2). The contents of TN, TP, and TK increased by 20.54, 83.98, and 12.08%, respectively, in the RP and PSB treatment compared with those in the RP only treatment.

### Soil Physicochemical Properties

According to the two-way ANOVA, the effects of RP, PSB, and their interaction on rhizosphere soil physicochemical properties were significant (*P* < 0.05) (Table 3). Compared with the untreated group (T0), the soil pH in T1, T0P, and T1P decreased by 0.13, 0.48, and 0.62 units, respectively, and the EC value in the PSB treatment group was lower than that in the treatment group without PSB. The treatments significantly affected the soil contents of Ca^2+^, Na^+^, and Cl^–^. The lowest Cl^–^ content was in the RP and PSB treatment groups. The treatments did not affect SO_4_^2–^ content.

**TABLE 3:** Effects of rock phosphate (RP) and phosphate-solubilizing bacteria (PSB) on soil physicochemical properties.

Treatment		pH	EC	Ca^2+^	Na^+^	Cl^–^	SO_4_^2–^
		
		–	μs/cm	g/kg	g/kg	g/kg	g/kg
-PSB	T0	7.96 ± 0.01^a^	1152.73 ± 39.84^b^	1.93 ± 0.02^c^	2.65 ± 0.01^a^	1.98 ± 0.02^a^	0.30 ± 0.01^b^
	T1	7.83 ± 0.02^b^	1281.53 ± 17.64^a^	2.85 ± 0.03^a^	0.13 ± 0.01^d^	0.35 ± 0.02^c^	0.37 ± 0.02^a^
+ PSB	T0P	7.48 ± 0.01^c^	1135.10 ± 11.22^d^	1.1 ± 0.02^d^	1.06 ± 0.01^b^	1.05 ± 0.01^b^	0.28 ± 0.01^c^
	T1P	7.34 ± 0.03^d^	1142.70 ± 27.69^c^	2.1 ± 0.03^b^	0.61 ± 0.01^c^	0.30 ± 0.01^c^	0.28 ± 0.01^c^

*Treatments: T0, no additions, the control (−PSB); T1, with RP amendment only (−PSB); T0P, with PSB inoculation only (+PSB); T1P, with RP and PSB (+PSB). EC, electrical conductivity. Values are the means of three replicates. Error bars represent ± standard deviation. Values sharing a common letter within the column are not significant at P < 0.05.*

**FIGURE 3:**
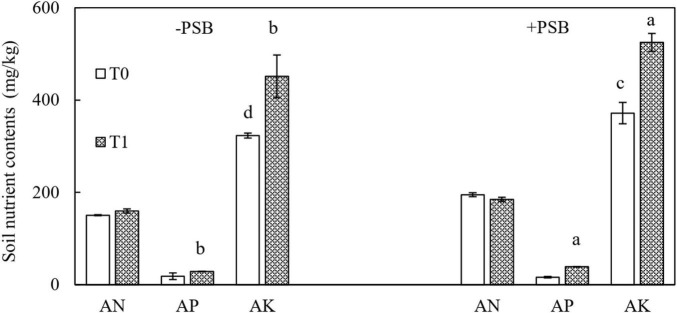
Effects of rock phosphate (RP) and phosphate-solubilizing bacteria (PSB) on soil available nitrogen (AN), available phosphorus (AP), and available potassium (AK) contents (mg/kg). Treatments: T0, without RP; T1, with RP; −PSB, without PSB; +PSB, with PSB. Values are the means of three replicates. Error bars represent ± standard deviation. Values sharing a common letter within the column are not significant at *P* < 0.05.

The effects of treatments on available nutrients in soil are shown in Figure 3. In the RP and PSB treatment, the contents were 184.90 mg/kg AN, 39.00 mg/kg AP, and 524.99 mg/kg AK. The difference was significant compared with no treatment, and the largest increase in soil AP (140.59%) was in the combined RP and PSB treatment.

**FIGURE 4:**
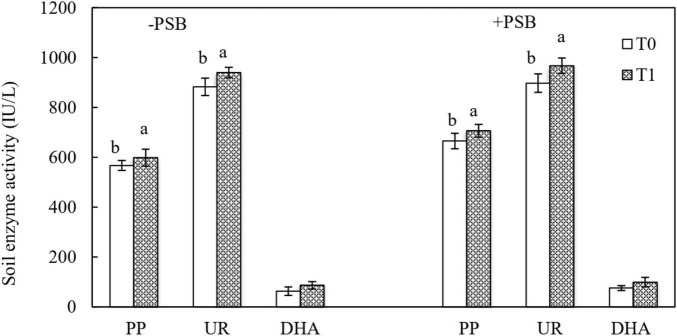
Effects of rock phosphate (RP) and phosphate-solubilizing bacteria (PSB) on soil alkaline phosphomonoesterases (PP), urease (UR), and dehydrogenase (DHA) enzyme activities (IU/L). Treatments: T0, without RP; T1, with RP; −PSB, without PSB; +PSB, with PSB. Values are the means of three replicates. Error bars represent ± standard deviation. Values sharing a common letter within the column are not significant at *P* < 0.05.

Figure 4 shows the activity of the soil enzymes PP, UR, and DHA. Compared with uninoculated soil, inoculation with PSB increased UR activity by 2.61% (T0P) and 2.82% (T1P). The highest PP activity of 706.54 IU/L was in the RP and PSB treatment. Dehydrogenase had the lowest activity, and the differences were not significant between inoculated and uninoculated soils in all treatments.

### Soil Microbial Community Diversity

This study is the first to investigate the effects of RP with or without PSB inoculation on the rhizosphere bacterial community of peanut (Table 4). The raw pyrosequencing data are accessioned at the NCBI SRA database with the BioProject accession number PRJNA777755. According to MiSeq analysis of the V3–V4 region of the bacterial 16S rRNA gene, the mean numbers of OTUs were 3,142 in T0, 2,879 in T1, 3,023 in T0P, and 2,674 in T1P. The mean Chao 1 and ACE indices were in the order T0 > T1 > T0P > T1P, indicating the level of community richness varied among the four treatments. The mean Shannon index indicated bacterial community diversity increased with PSB inoculation. The index ranged from 9.30 to 9.65 with PSB inoculation but from 8.92 to 8.97 without PSB inoculation. The results were similar for the Simpson index.

**TABLE 4:** Effects of rock phosphate (RP) and phosphate-solubilizing bacteria (PSB) on richness and diversity indices of soil bacterial communities.

Sample	Observed_species	Chao1	ACE	Shannon	Simpson
T0	3142.33 ± 43.99^a^	4063.15 ± 111.21^a^	4061.77 ± 54.96^a^	8.97 ± 0.02^c^	0.98 ± 0.00^b^
T1	3029.33 ± 177.34^b^	3903.36 ± 230.17^b^	3911.65 ± 217.91^b^	8.92 ± 0.18^c^	0.99 ± 0.00^a^
T0P	3023 ± 103.41^b^	3549.44 ± 241.13^c^	3578.69 ± 207.97^c^	9.65 ± 0.32^a^	0.99 ± 0.00^a^
TIP	2674 ± 130.23^c^	3262.13 ± 277.50^d^	3227.01 ± 312.94^d^	9.30 ± 0.0^b^	0.99 ± 0.00^a^

*Treatments (following the treatment code is the replicate number): T0, no additions, the control; T1, with RP amendment only; T0P, with PSB inoculation only; T1P, with RP and PSB. Values sharing a common letter within the column are not significant at P < 0.05.*

**FIGURE 5:**
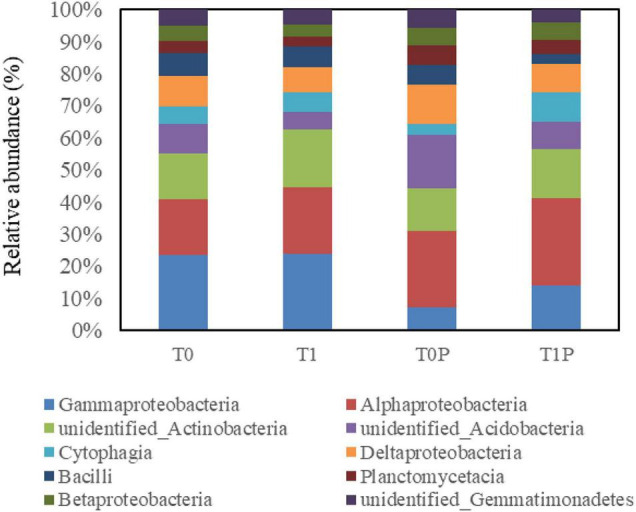
Effects of rock phosphate (RP) and phosphate-solubilizing bacteria (PSB) on the relative abundance (%) of the 10 dominant classes of soil bacteria. The same 10 classes were dominant in all treatments. Treatments (following the treatment code is the replicate number): T0, no additions, the control; T0P, with PSB inoculation only; T1, with RP amendment only; T1P, with RP and PSB.

All valid sequences from the soil sample libraries were classified at the class and genus levels. At the class level, the top ten dominant classes were selected (Figure 5). The 10 dominant bacterial classes were Gammaproteobacteria, Alphaproteobacteria, Actinobacteria, Acidobacteria, Cytophagia, Deltaproteobacteria, Bacilli, Planctomycetacia, Betaproteobacteria, and Gemmatimonadetes. The dominant classes were the same in different treatments; however, the relative abundance of each class was different. The relative abundance of Alphaproteobacteria, Betaproteobacteria, and Deltaproteobacteria was higher in rhizosphere soils with PSB inoculation than in those without. By contrast, the relative abundance of Gammaproteobacteria was higher in soils without PSB inoculation.

**FIGURE 6:**
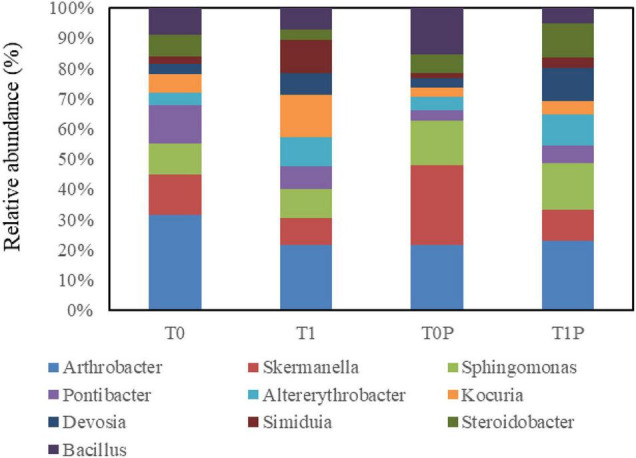
Effects of rock phosphate (RP) and phosphate-solubilizing bacteria (PSB) on the relative abundance (%) of the 10 dominant genera of soil bacteria. The same 10 genera were dominant in all treatments. Treatments (following the treatment code is the replicate number): T0, no additions, the control; T0P, with PSB inoculation only; T1, with RP amendment only; T1P, with RP and PSB.

Figure 6 shows the relative abundance of bacteria at the genus level. The 10 dominant genera were *Arthrobacter*, *Skermanella*, *Sphingomonas*, *Pontibacter*, *Altererythrobacter*, *Kocuria*, *Devosia*, *Simiduia*, *Steroidobacter*, and *Bacillus*. The total abundance of the 10 dominant bacterial genera without PSB inoculation (T0, T1) was higher than that with inoculation (T0P, T1P). The lowest total abundance of the 10 dominant genera of bacteria was in T0P, accounting for 10.64% of the total bacteria. The total abundance in T1 and T1P accounted for 18.42 and 17.48% of total bacteria, respectively. Therefore, the addition of PR and PSB greatly affected the relative abundance of the dominant genera of bacteria.

**FIGURE 7:**
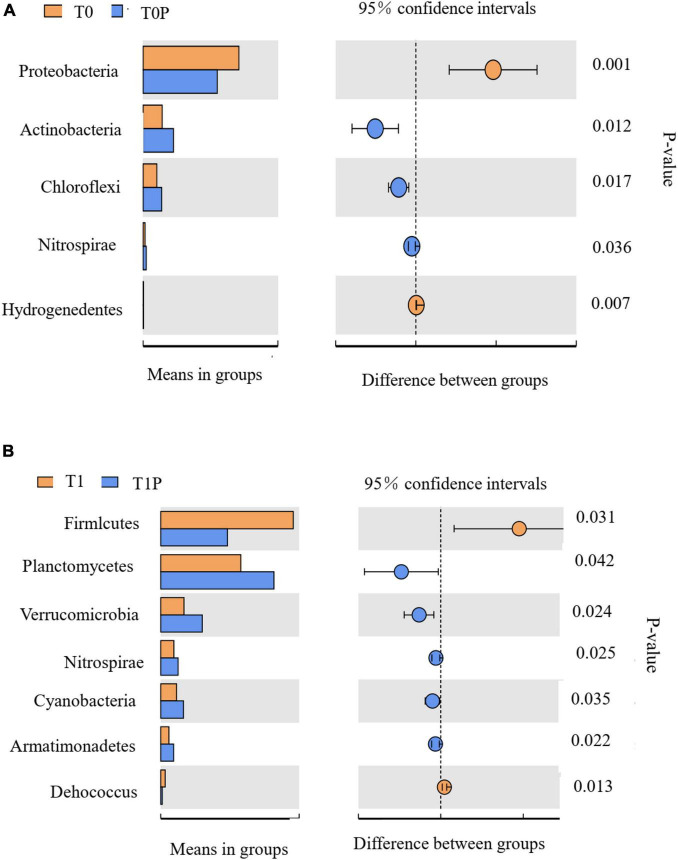
Bacterial phyla with relative abundance significantly different in the comparison between **(A)** control soil with no additions (T0) and soil with only phosphate-solubilizing bacteria (PSB) (T0P) and between **(B)** soil with only rock phosphate (RP) amendment (T1) and soil with both RP and PSB (T1P). Significant differences in relative abundance were determined by *t*-tests. On the left: the abundance of taxa in each group, with each horizontal bar representing the mean value. On the right: the intergroup difference confidence level and the *P*-value for the significant difference in abundance.

Figure 7 shows comparisons between two groups: T0 vs. T0P (Figure 7A) and T1 vs. T1P (Figure 7B). Within each comparison, significant differences in bacterial groups were determined using *t*-tests. Compared with T0, *Acidobacteria*, *Chloroflexi*, and *Nitrospirae* were more important in T0P (*P* < 0.05). By contrast, *Proteobacteria* and *Hydrogenedentes* were more important in T0. Compared with T1, *Planctomycetes*, *Verrucomicrobia*, *Nitrospirae*, *Cyanobacteria*, and *Armatimonadetes* were more important in T1P. Thus, PSB inoculation significantly affected those taxa. In all treatments, *Acidobacteria*, *Chloroflexi*, and *Planctomycetes* were enriched after inoculation with PSB.

Pearson’s coefficients of correlation (*r*) and significance (*P*) values were calculated between the most abundant bacterial phyla and peanut growth parameters (Table 5) and soil properties (Table 6). The relative abundance of *Planctomycetes* was positively correlated with root length, and the relative abundances of *Acidobacteria*, *Chloroflex*, and *Planctomycetes* were positively correlated with root dry weight (*P* < 0.05; Table 5). Therefore, the three bacterial phyla likely had positive effects nutrient uptake and growth in peanut. The relative abundance of *Firmicutes* was negatively correlated with AP and AK and positively correlated with soil pH (Table 6). *Planctomycetes* was positively correlated with AN and negatively correlated with soil EC (*P* < 0.05). *Acidobacteria* and *Chloroflexi* were also positively correlated with AN. alkaline phosphomonoesterases, UR, and DHA activities were not correlated with the relative abundance of the bacterial phyla (*P* > 0.05). The results showed that PSB application significantly increased soil bacterial populations and favored those phyla that were associated with soil nutrient cycling.

**TABLE 5:** Pearson’s coefficients of correlation (*r*) between the 10 most abundant phyla of soil bacteria and measures of peanut growth.

Phylum	RL	SL	RFW	SFW	SDW	RDW
	
	(cm)	(cm)	(g/plant)–	(g/plant)	(g/plant)	(g/plant)
*Proteobacteria*	–0.46	–0.09	0.14	–0.13	–0.32	**−−0.54**
*Actinobacteria*	–0.07	–0.31	0.31	–0.11	0.37	–0.14
*Acidobacteria*	0.36	0.23	–0.36	0.14	0.17	**0.69**
*Bacteroidetes*	–0.10	0.24	0.20	0.05	–0.37	**−−0.5**
*Chloroflexi*	0.25	–0.10	–0.37	0.02	0.21	**0.62**
*Firmicutes*	–0.13	–0.47	0.18	–0.26	0.11	–0.17
*Planctomycetes*	**0.59**	0.28	–0.08	0.32	0.17	**0.51**
*Gemmatimonadetes*	–0.27	–0.07	–0.47	–0.13	–0.16	0.1
*Thaumarchaeota*	0.30	–0.02	0.16	0.02	0.15	0.28
*Thermomicrobia*	0.34	–0.06	0.18	0.14	0.39	0.37

*RL, root length; SL, stem length; RFW, root fresh weight; SFW, stem fresh weight; SDW, stem dry weight; RDW, root dry weight. Bold type indicates a significant correlation (p < 0.01).*

**TABLE 6:** Pearson’s coefficients of correlation (*r*) between the 10 most abundant phyla of soil bacteria and soil chemical properties and enzyme activities.

Phylum	pH	EC	AN	AP	AK	PP	UR	DHA
	
		(μs/cm)	(mg/g)	(mg/g)	(mg/g)	(IU/L)	(IU/L)	(IU/L)
*Proteobacteria*	0.26	0.29	**−−0.54**	–0.05	0.16	0.35	0.13	–0.07
*Actinobacteria*	0.21	0.30	–0.15	–0.13	–0.03	–0.21	0.04	0.13
*Acidobacteria*	–0.37	–0.38	**0.66**	0.21	–0.09	–0.16	–0.08	0.04
*Bacteroidetes*	–0.16	0.10	–0.28	0.23	0.32	0.45	0.22	0.29
*Chloroflexi*	–0.16	–0.33	**0.46**	–0.04	–0.22	–0.39	–0.14	–0.07
*Firmicutes*	**0.71**	0.19	–0.44	**−−0.63**	**−−0.54**	–0.47	–0.47	–0.63
*Planctomycetes*	–0.67	**−−0.57**	**0.75**	0.47	0.22	0.15	0.08	0.25
*Gemmatimonadetes*	0.09	0.08	–0.03	–0.08	–0.15	–0.03	–0.05	–0.15
*Thaumarchaeota*	–0.07	–0.32	0.35	–0.13	–0.28	–0.40	–0.31	–0.16
*Thermomicrobia*	0	–0.16	0.31	–0.17	–0.26	–0.44	–0.19	–0.14

*EC, electrical conductivity; AN, available nitrogen; AP, available phosphorus; AK, available potassium; PP, alkaline phosphomonoesterases activity; UR, urease activity; DHA, dehydrogenase activity.*

*Bold type indicates a significant correlation (p < 0.01).*

## Discussion

Saline soils negatively affect plant growth because of nutrient deficiencies and salts (Yang et al., 2012). To address these problems, the combined application of RP and PSB as a potential substitute for chemical fertilizers has aroused considerable recent attention (Yadav et al., 2017). PSB play an important role in P cycling in soil–plant systems. Many PSB strains in the genera *Burkholderia, Enterobacter, Paenibacillus*, and *Pseudomonas* can potentially promote plant growth, by participating in various biological processes (Ait Barka et al., 2006). Shahzad et al. (2017) reported that the interaction between RP and PSB improved soil biochemical properties in dryland agriculture. However, the current study is the first to report that the interaction between RP and halotolerant PSB (*Providencia rettgeri* strain TPM23) improved soil biochemical properties and peanut production in a saline soil.

The application potential of halotolerant PSB in agriculture is based on their ability to increase crop growth and eliminate the effects of stress without harming the environment (Shukla et al., 2012). The addition of P fertilizer can strengthen the effects of PSB because it provides a substrate that benefits the proliferation and survival of inoculated PSB (Yu et al., 2011). In this study, the growth of peanut in a saline soil increased under the combined the effect of RP and PSB. Other studies also indicate that PSB combined with an insoluble phosphate source can improve plant growth and yield as well as soil conditions (Gurdeep and Reddy, 2015; Mahanta et al., 2018). Estrada et al. (2013) and Yadav et al. (2014) found similar results with tricalcium phosphate or P_2_O_5_ amendments. Phosphorus uptake by plants reflects P availability in various treatments. In the present study, the combined application of PSB and RP significantly increased P uptake in comparison with only RP amendment or PSB inoculation. In addition, for the treatments with combined applications of PSB and RP with an increase in peanut growth parameters, N, P, and K accumulation increased significantly in leaves. Similarly, Khosravi et al. (2018) found that the co-application of plant growth-promoting rhizobacteria (PGPR) and RP significantly increased N, P, and K uptake rates in lettuce, which led to increases in biomass and length. Thus, the inoculation of seeds with PSB combined with a mineral P source is beneficial to crop growth, regardless of the type of mineral P. The application of RP provides a nutrient substrate for PSB, and a large population of PSB can continuously dissolve the RP and release much more available P for plants, As a result, plant growth improves and biomass increases.

In addition to effects on plants, the complex interaction between PSB and RP also affects soil physicochemical properties. One of the main mechanisms leading to the dissolution of inorganic phosphate in soil is the reduction in rhizosphere pH due to the release organic acids (Rfaki et al., 2020). A small reduction in soil pH was observed with PSB inoculation or RP amendment compared with that in the control. However, the most significant reduction in pH was in the combined PSB and RP treatment. The greater reduction in pH might be because the RP was a substrate for PSB and associated with subsequent increases in microbial biomass would be increased excretion of organic acids, lowering the pH. The decrease in rhizosphere pH would then lead to the dissolution of P sources and increase the availability of P (Taktek et al., 2017). Moreover, according to Arora (2016), PSB can be used to decrease or exclude soil ions. Similarly, we found that Cl^–^ and Na^+^ decreased in the combined RP and PSB treatment. The decrease might be because the Ca^2+^ of RP can react with Na^+^ to form a calcium colloid, which increases the formation of soil aggregates and improves soil structure (Lebron et al., 2002; Chen et al., 2009). The solubility of the Na_2_SO_4_ is also greatly reduced compared with that of NaCl, which increases the salt-leaching of salinity components and inhibits soil processes. Thus, the application of RP and PSB reduced the pH while optimizing the salt–alkali ion composition. However, the content of Ca^2+^ increased with the addition of RP, likely because it was enriched with calcium ions.

Additionally, PGPR are exogenous bacteria introduced into soils to participate in soil processes such as the storage and release of nutrients (Ribeiro and Cardoso, 2012; Patel and Saraf, 2013). In this study, we found a higher level of AN, AP, and AK with PSB inoculation. Da Costa et al. (2015) also found a significantly higher level of AP when RP was combined with PSB, compared with other treatments. The PSB acidify soil by producing organic acids, which increases the solubility of P from RP (Yadav et al., 2015). In addition, PSB increases alkaline phosphomonoesterases activity to increase the solubility of P (Prabhu et al., 2018). In this study, the addition of PSB significantly affected soil enzyme activity. This result may have been related to the number and diversity of soil microorganisms, and that the metabolic activity of microorganisms can enhance soil enzyme activity. The secretion of organic acids and an increase in the root exudates can also increase soil enzyme activity. This result is consistent with that of Balota and Chaves (2010) who found that increases in soil enzyme activity are related to PGPR inoculation.

Soil microorganisms are critical to ecosystem functions and the maintenance of soil fertility. However, in saline soils, microbial populations are often restricted by nutrient deficiencies. Plant growth-promoting rhizobacteria (PGPR) are exogenous bacteria, and inoculation with PGPR can greatly affect existing soil microbial communities (Jeong et al., 2013; Bharti et al., 2015; Sheridan et al., 2017). In this study, PSB inoculation strongly increased the α-diversity of bacterial communities. Such enrichment has been attributed to increased dissolution of chemical substrates with bio-inoculation, because released nutrients are then used by resident populations to promote their growth (Bharti et al., 2015). However, the distribution of sequences demonstrated that each rhizosphere bacterial community was unique, indicating that PSB inoculation stimulated the growth of additional bacterial taxa. In this study, the phyla *Proteobacteria*, *Acidobacteria*, *Actinobacteria*, *Chloroflexi*, *Bacteroidetes*, *Planctomycetes*, and *Gemmatimonadetes* dominated bacterial communities. Li et al. (2016) also found that most of these bacterial phyla were dominant in saline soils. However, the structure of rhizosphere-associated bacterial communities was different among treatments in this study. The application of RP and inoculation with PSB, as well as their interaction, were responsible for the differences. These results are consistent with those of Silva et al. (2017) who found an increase in bacterial communities in soils with long-term RP fertilization. Bacterial community structure was different between treatments with and without PSB inoculation. The differences might be because PSB inoculation was the major factor in determining the structure of bacterial communities in the saline soil. *Acidobacteria*, *Chloroflexi*, and *Planctomycetes* were most affected by treatment and were the dominant bacterial phyla in the combined RP and PSB treatment (*P* < 0.05). In addition, the three phyla were significantly positively correlated with root dry weight. Thus, the inoculation of PSB combined with RP favored the establishment of three dominant bacterial phyla and further promoted peanut growth. The phylum *Acidobacteria* is important in nutrient turnover and is also highly involved in rhizosphere physiological processes. In addition, the abundance of the *Planctomycetes* increases strongly following additions of inorganic P (Ganzert et al., 2011).

Luo et al. (2017) analyzed the functional genes and bacterial community structures associated with the P cycle and found that bacteria with the alkaline phosphomonoesterases gene *phoD* in soil are mainly composed of *Actinobacteria* and *Proteobacteria*. In this study, *Actinobacteria* were enriched after inoculation with PSB, which could lead to increases in alkaline phosphomonoesterases activity and thereby increase the availability of P in the saline soil. All the above results showed that the amendments affected the abundance and diversity of bacterial communities The application of PSB significantly increased the abundance of bacteria associated with soil nutrient cycling processes, which ultimately affects the health of saline soil.

## Conclusion

In this study, halotolerant PSB (*Providencia rettgeri* strain TPM23) and RP effectively alleviated salt stress in peanut in saline soil. A strong synergistic effect was found between PSB inoculation and RP amendment for promoting plant growth and soil quality as well as microbial community diversity. The application of PSB significantly increased the populations of *Acidobacteria*, *Chloroflexi*, and *Planctomycetes*, which are phyla involved in nutrient cycling processes that could ultimately affect the health of the saline soil. Therefore, the combination of PSB and RP might be a low-cost and environmentally safe alternative strategy to remediate the problem of low nutrient availability in saline soils.

## Data Availability Statement

The datasets presented in this study can be found in online repositories. The names of the repository/repositories and accession number(s) can be found below: https://www.ncbi.nlm.nih.gov/genbank/, KX834965.

## Author Contributions

HJ and PQ designed the research. HJ and TW performed the experiment and data analysis. HJ wrote the manuscript. GC and SL participated in data download, manuscript revision, and document proofreading. XC guided the research. All the authors had read, edited and approved the final manuscript.

## Conflict of Interest

The authors declare that the research was conducted in the absence of any commercial or financial relationships that could be construed as a potential conflict of interest.

## Publisher’s Note

All claims expressed in this article are solely those of the authors and do not necessarily represent those of their affiliated organizations, or those of the publisher, the editors and the reviewers. Any product that may be evaluated in this article, or claim that may be made by its manufacturer, is not guaranteed or endorsed by the publisher.

## Footnotes

1
http://qiime.org/index.html
